# Therapeutic Approaches for Enhancing Spinal Fusion in Low Back Pain: A Review With a Focus on the Elderly

**DOI:** 10.1002/jsp2.70136

**Published:** 2025-11-12

**Authors:** Shuimu Chen, Zhen Li, Sebastian F. Bigdon, Sonja Häckel, Christoph E. Albers, Benjamin Gantenbein

**Affiliations:** ^1^ Tissue Engineering for Orthopedics & Mechanobiology (TOM), Bone & Joint Program, Department for Bio‐Medical Research (DBMR), Faculty of Medicine University of Bern Bern Switzerland; ^2^ Graduate School for Cellular and Biomedical Sciences (GCB) University of Bern Bern Switzerland; ^3^ Department of Orthopedic Surgery & Traumatology, Inselspital University of Bern Bern Switzerland; ^4^ AO Research Institute Davos Davos Switzerland; ^5^ Graduate School for Health Sciences University of Bern Bern Switzerland

**Keywords:** artificial intelligence, bone homeostasis, low back pain, osteoporosis, precision medicine, spinal fusion, the elderly

## Abstract

**Background:**

Low back pain (LBP) is a prevalent cause of disability worldwide, particularly among the elderly, with degenerative spinal conditions often necessitating surgical intervention. Spinal fusion remains a definitive treatment for patients unresponsive to conservative therapies, yet its success is challenged by age‐related factors such as osteoporosis, diminished stem cell function, and vascular insufficiency.

**Methods:**

This review examines current and emerging strategies to improve spinal fusion outcomes for elderly patients by analyzing advances in biomaterials, growth factor delivery systems, cell‐based regenerative therapies, surgical innovations, and some novel approaches.

**Results:**

Advances in biomaterials, including bioactive scaffolds, 3D‐printed constructs, and hybrid grafts, provide structural and biological support for bone formation. Growth factor delivery systems, particularly controlled‐release formulations of bone morphogenetic proteins (BMPs) and vascular endothelial growth factor (VEGF), improve osteoinduction while mitigating adverse effects. Cell‐based regenerative therapies utilizing mesenchymal stromal cells (MSCs) and extracellular vesicles (EVs) offer promising osteogenic and immunomodulatory potential. Furthermore, minimally invasive surgical techniques and robotic‐assisted procedures provide additional options for enhancing spinal fusion in elderly patients. Novel approaches targeting cellular senescence, epigenetic modulation, and mitochondrial dysfunction are emerging to counteract age‐related impairments in bone formation.

**Conclusion:**

Despite significant advancements, challenges such as optimizing biomaterial integration, mitigating inflammatory responses, and ensuring long‐term stability remain. Future research should leverage precision medicine, artificial intelligence, and nanotechnology to enable patient‐specific fusion strategies. A multidisciplinary approach will be essential to improve spinal fusion outcomes for aging populations.

## Graphical abstract

The review summarizes the current clinical problems of non‐spinal fusions in orthopaedics. I refers to selected biological examples of how ossification can be better achieved in elderly patients.

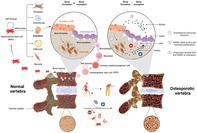

## 1 Introduction

Low back pain (LBP) is a pervasive health issue, particularly among the elderly population, and is a leading cause of disability worldwide. It is estimated that approximately 60% of individuals will experience LBP at some point in their lives, with the prevalence increasing significantly with age [1]. Degenerative spinal conditions, such as lumbar spinal stenosis, intervertebral disc degeneration, and spondylolisthesis, are common contributors to LBP in the elderly (≥ 60 years) [2]. For patients who fail to respond to conservative treatments, spinal fusion surgery is often considered a definitive solution to stabilize the spine, alleviate pain, and improve functional outcomes. In this procedure, motion segments of the spine are surgically immobilized by placing bone grafts or substitutes between vertebrae, usually in the lumbar region, with or without the aid of instrumentation (e.g., pedicle screws and rods) [3]. The ultimate goal is to achieve solid arthrodesis across the targeted vertebral levels [4]. However, the success of spinal fusion is highly dependent on the patient's ability to achieve solid bone union, a process that becomes increasingly challenging with advancing age [5].

The elderly population presents unique challenges for spinal fusion due to age‐related physiological changes. Osteoporosis, a condition characterized by reduced bone density and quality, is prevalent among older adults and significantly impairs bone formation capacity. Additionally, the decline in stem cell function, reduced vascularization, and the presence of comorbidities such as diabetes and cardiovascular diseases could further complicate the fusion process [6, 7, 8]. These factors collectively contribute to relatively high rates of pseudarthrosis and postoperative complications in elderly patients, underscoring the need for innovative strategies to enhance spinal fusion outcomes in this demographic.

Recent advancements in biomaterials, growth factors, and cell‐based therapies have provided new insights into addressing these challenges [9, 10, 11, 12]. However, the translation of these technologies into clinical practice requires a thorough understanding of the biological and mechanical principles underlying spinal fusion, as well as the specific needs of elderly patients. This review aims to provide a comprehensive overview of current and emerging therapeutic approaches for enhancing spinal fusion, with a particular focus on the elderly population.

## 2 Principles and Challenges of Spinal Fusion

### 2.1 Biological Mechanisms of Spinal Fusion

Spinal fusion is a complex biological process that mimics natural bone formation, involving three overlapping phases: inflammation, repair, and remodeling. During the inflammatory phase, hematoma formation and the recruitment of immune cells create a microenvironment conducive to bone formation. The repair phase is characterized by the formation of a soft callus, which is gradually replaced by hard callus through endochondral ossification. Finally, the remodeling phase involves the maturation and reorganization of the newly formed bone to achieve mechanical stability [13].

Key cellular players in this process include osteoblasts (OBs), osteoclasts (OCs), and mesenchymal stromal cells (MSCs). OBs are responsible for bone formation, while OCs mediate bone resorption, ensuring a balanced remodeling process. MSCs, which differentiate into OBs, play a critical role in initiating and sustaining bone formation. Growth factors such as bone morphogenetic proteins (BMPs), platelet‐derived growth factor (PDGF), and vascular endothelial growth factor (VEGF) are essential for regulating these cellular activities and promoting successful fusion [14]. In addition, the role of the intervertebral disc in interbody fusion should not be overlooked. The disc secretes several BMP antagonists, including Noggin, Gremlin 1, and Chordin, which can bind to BMP2 and inhibit its osteoinductive activity, thereby potentially exerting a negative influence on the fusion process [15, 16, 17]. Therefore, careful and thorough removal of the affected intervertebral disc during operation may help reduce this inhibitory effect on spinal fusion.

### 2.2 Challenges in Elderly Patients

Elderly patients face several age‐related challenges that could hinder the success of spinal fusion. Osteoporosis, a hallmark of aging, not only reduces bone density but also alters bone microarchitecture, making it more susceptible to fractures and impairing the integration of fusion constructs, as well as the subsidence of intervertebral body devices [18]. The decline in MSC populations and their reduced differentiation potential further compromise bone formation capacity [19]. Additionally, age‐related changes in the vascular system, such as reduced blood flow and angiogenesis, limit the supply of nutrients and oxygen necessary for bone formation [20].

Comorbidities, such as diabetes and cardiovascular diseases, are common in the elderly and exacerbate challenges in spinal fusion surgery. Diabetes, for instance, is associated with impaired OB function and delayed bone formation, while cardiovascular diseases can reduce perfusion to the surgical site [21, 22]. Furthermore, the use of medications such as glucocorticoids and proton pump inhibitors, which are frequently prescribed to older adults, can negatively impact bone metabolism and fusion outcomes [23, 24].

**FIGURE 1:**
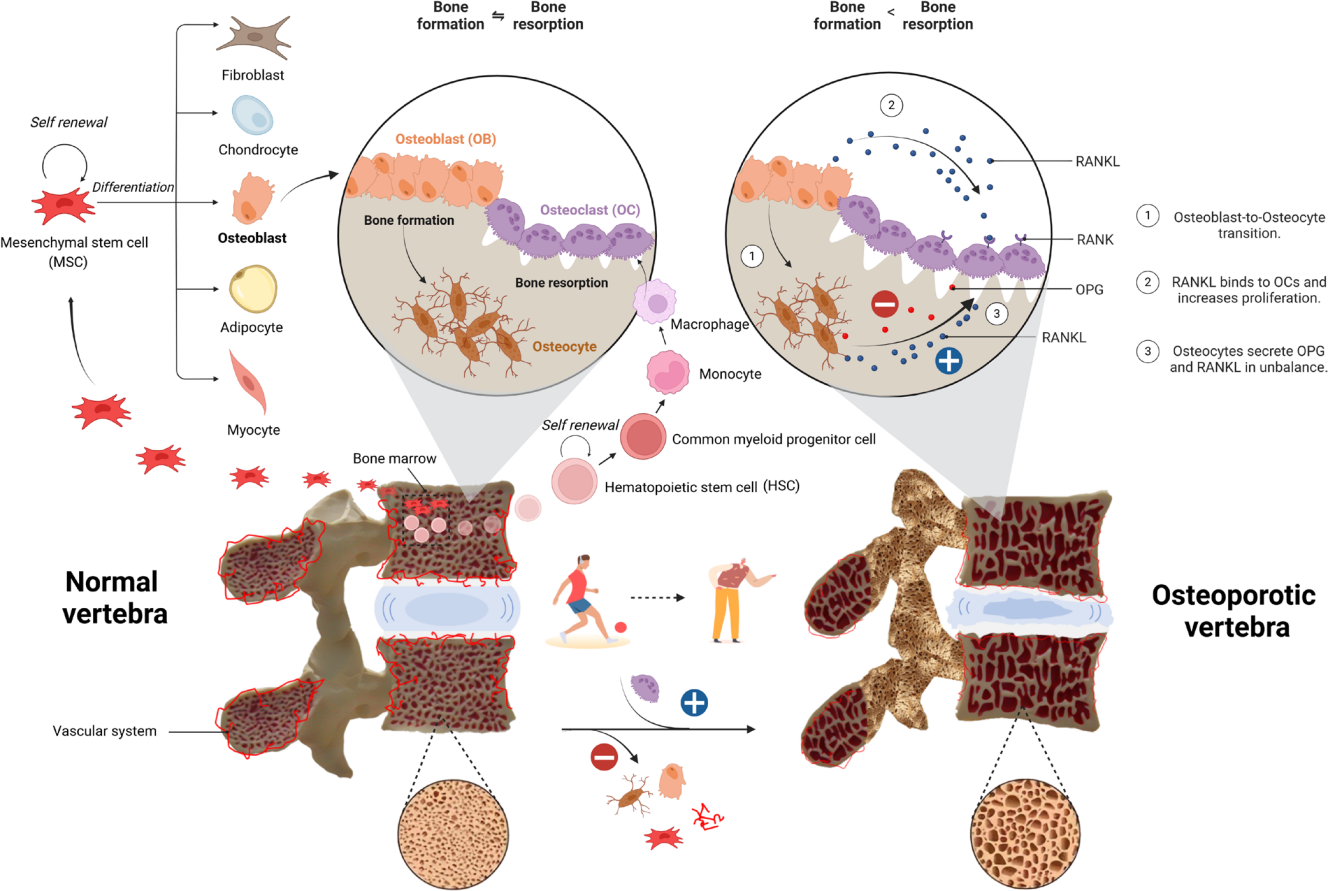
Bone homeostasis and osteoporosis‐related challenges in spinal fusion in the vertebrae. This figure illustrates the key biological and mechanical challenges affecting spinal fusion in elderly patients. In normal vertebrae, MSCs within the bone marrow differentiate into osteoblasts, which mature into osteocytes and maintain bone formation. Concurrently, hematopoietic stem cells (HSCs) differentiate into OCs, which mediate bone resorption. Under normal vascular conditions, bone formation and resorption remain balanced. In aged vertebrae, MSCs exhibit impaired osteogenic differentiation, coupled with reduced vascular supply, leading to decreased bone formation. Meanwhile, osteoblasts and osteocytes secrete increased levels of RANKL (receptor activator of nuclear factor kappa‐B ligand), disrupting the OPG (osteoprotegerin)/RANKL balance and excessively activating OCs, resulting in accelerated bone resorption. Ultimately, osteoporosis and degenerative changes in the intervertebral disc contribute to the structural fragility of the aged spine.

Mechanical stability is a critical determinant of spinal fusion success. In elderly patients, the reduced bone quality often necessitates the use of additional instrumentation, such as pedicle screws with cement augmentation and rods, to achieve adequate stabilization. However, the increased rigidity of these constructs can lead to stress shielding, where the lack of mechanical loading on the fusion site inhibits bone formation [25]. Balancing mechanical stability with the promotion of biological healing remains a significant challenge in spinal fusion surgery for the elderly (Figure 1).

## 3 Current Strategies for Enhancing Spinal Fusion

Spinal fusion success hinges on the interplay of biological, mechanical, and pharmacological interventions. Current strategies aim to address critical challenges such as osteogenic insufficiency, biomechanical instability, and age‐related comorbidities. These challenges necessitate a multidisciplinary approach that incorporates advances in biomaterials, cellular therapies, growth factor delivery, and surgical innovations to optimize fusion outcomes and reduce complications.

### 3.1 Bone Grafts and Biomaterials

Autologous bone grafts, particularly those harvested from the iliac crest or collected intraoperatively from decompression or corpectomy procedures, represent a good source of bone material that is often superior in clinical application. They remain the clinical gold standard for spinal fusion due to their intrinsic osteogenic, osteoinductive, and osteoconductive properties [26, 27, 28]. Unlike iliac crest grafts, these intraoperative bone fragments are naturally available during surgical exposure and debridement, eliminating the need for an additional donor site and thereby reducing the risk of donor site morbidity. Moreover, the utilization of these bone fragments minimizes material wastage and optimizes resource efficiency, as these elements would otherwise be discarded. The incorporation of such autologous sources can enhance fusion outcomes by preserving the patient's native biological properties while simultaneously mitigating complications associated with secondary surgical harvesting. However, the literature suggests that autologous local bone obtained intraoperatively may have a lower osteogenic potential compared to iliac crest bone grafts [29]. This may be due to a smaller volume of available graft, a higher content of fibrous and cortical tissue, and a reduced proportion of cancellous bone. Intraoperative autologous bone fragments are biologically favorable and eliminate donor site morbidity, but their effectiveness in elderly patients warrants critical consideration. Age‐related bone loss and osteoporotic changes can compromise both the biological activity and mechanical integrity of cancellous bone harvested intraoperatively. Studies have shown that osteoporotic bone exhibits reduced osteogenic cell numbers, diminished osteoinductive signaling, and impaired vascularization, all of which may attenuate the graft's regenerative potential [30, 31]. Moreover, the weakened trabecular architecture and lower bone mineral density in elderly patients may reduce the mechanical performance of such grafts, limiting their ability to provide stable structural support [30]. Therefore, although intraoperative autografts remain a valuable resource, their limitations in older adults highlight the need for adjunctive strategies, such as the incorporation of osteoinductive agents or the supplementation with bone substitutes, to optimize fusion outcomes in this population.

Allografts, while exhibiting reduced immunogenicity compared with xenografts, often display diminished osteoinductive capacity [32, 33]. Therefore, augmentation with bioactive molecules such as BMP‐2 or PDGF has been explored to enhance bone formation [34, 35]. In addition to these biological enhancements, recent studies have investigated the incorporation of demineralized bone matrix (DBM) and bone marrow aspirate concentrate (BMAC) to further improve osteoinductive potential, providing a more effective scaffold for cellular infiltration and bone formation [26, 36, 37]. Synthetic bone substitutes, including hydroxyapatite (HA) and β‐tricalcium phosphate (β‐TCP), offer a biocompatible and structurally stable alternative to biological grafts [38, 39]. Other advanced biomaterials such as bioactive glass, calcium sulfate, and magnesium‐based scaffolds have gained attention for their ability to actively participate in the bone remodeling process, promoting osteoconduction and angiogenesis [40, 41, 42]. Additionally, polymer‐based biomaterials, such as polyetheretherketone (PEEK) and polylactic‐co‐glycolic acid (PLGA), are being explored for their biomechanical compatibility and potential for drug delivery applications in spinal fusion [43, 44]. Recent studies have explored hybrid biomaterials that integrate synthetic scaffolds with bioactive molecules, such as BMP‐2 and collagen‐binding peptides, to mimic the natural bone extracellular matrix (ECM) and enhance osteogenic potential [45, 46, 47, 48].

Three‐dimensional (3D) printing has emerged as a groundbreaking technology in biomaterial engineering, revolutionizing the development of patient‐specific scaffolds with optimized architectural, mechanical, and biological properties [49, 50, 51]. This technology enables precise control over scaffold porosity, interconnectivity, and mechanical integrity, allowing for the creation of highly biomimetic structures that closely resemble native bone tissue [52, 53]. Recent advancements have focused on 3D‐printed porous titanium and bioresorbable polymeric scaffolds, functionalized with osteoinductive proteins such as BMP‐2 and collagen‐binding peptides, to enhance osteogenesis and accelerate spinal fusion [54, 55, 56]. Furthermore, incorporating bioactive ceramic coatings, including HA and calcium phosphate, has been shown to improve osseointegration by promoting direct bone apposition and cellular attachment [38, 57]. Additionally, hybrid scaffolds combining metallic frameworks with biodegradable polymers provide a balance between immediate mechanical support and gradual bioresorption, aligning with the dynamic remodeling of the fusion site [58]. These innovations in 3D‐printed biomaterials hold significant potential for advancing personalized spinal fusion treatments by tailoring implants to patient‐specific anatomical and biomechanical needs, ultimately improving clinical outcomes and reducing complication rates.

### 3.2 Growth Factor Delivery Systems

Growth factors play a pivotal role in orchestrating cellular processes that are essential for effective bone formation and spinal fusion. Among these, recombinant BMPs, particularly BMP‐2 and BMP‐7, have demonstrated strong osteoinductive properties, making them highly valuable in clinical applications [26, 59]. However, their use at supraphysiological doses has been associated with significant adverse effects, including ectopic bone formation, radiculitis, and cyst formation, which limit their broad applicability [26]. To address these concerns, recent research efforts have focused on refining delivery mechanisms that optimize the localized, sustained release of BMP‐2, thereby reducing systemic exposure and potential complications.

Innovative delivery platforms based on nanotechnology, such as heparin‐conjugated hydrogels and nanoparticle carriers, as well as related microscale systems like biodegradable microparticles, have been designed to ensure controlled and site‐specific release of growth factors [60, 61, 62, 63, 64]. These systems not only enhance the bioavailability of growth factors but also minimize off‐target effects, reducing the risks associated with high systemic doses [65]. Furthermore, advanced biomaterials incorporating stimuli‐responsive drug release mechanisms, such as pH‐sensitive or enzyme‐degradable matrices, allow for dynamic regulation of BMPs bioactivity, aligning with the natural healing phases of bone formation [66, 67]. In addition to BMPs, alternative growth factors such as VEGF and fibroblast growth factor‐2 (FGF‐2) are being actively investigated for their complementary roles in bone repair. VEGF plays a crucial role in promoting angiogenesis, ensuring sufficient blood supply to the fusion site, while FGF‐2 enhances osteoprogenitor cell recruitment and proliferation [68, 69, 70]. The synergistic effects of these growth factors create a more conducive microenvironment for bone formation, particularly in compromised conditions such as osteoporosis or hypovascular environments often observed in elderly patients. As a result, combinatorial approaches integrating multiple growth factors within advanced biomaterial carriers are emerging as promising strategies to enhance spinal fusion outcomes.

### 3.3 Cell‐Based Regenerative Therapies

The advent of regenerative medicine has catalyzed extensive research into MSCs‐based therapies for spinal fusion. MSCs possess multipotent differentiation capabilities and exert profound effects through paracrine signaling, facilitating osteogenesis, angiogenesis, and modulation of inflammatory responses [71, 72, 73]. Notably, MSCs‐seeded scaffolds, particularly those enriched with osteoinductive cues such as mechanical stimulation, hypoxia preconditioning, or controlled bioreactor‐based expansion, have demonstrated remarkable efficacy in promoting fusion mass formation [74, 75, 76, 77]. These preconditioning strategies enhance MSCs' survival, proliferation, and osteoblastic differentiation, optimizing the regenerative potential of the graft.

Emerging research has also underscored the promise of MSCs‐derived extracellular vesicles (EVs) as acellular substitutes for direct MSCs transplantation. These EVs carry a diverse array of bioactive molecules, including non‐coding RNAs such as microRNAs (miRNAs), long non‐coding RNAs (lncRNAs), and circular RNAs (circRNAs), all of which play critical roles in modulating osteogenic differentiation and spinal fusion [78, 79, 80, 81, 82]. EVs serve as key mediators of intercellular communication, encapsulating osteogenic microRNAs (e.g., miR‐29b, miR‐148a), lncRNAs, circRNAs, and critical osteogenic proteins such as alkaline phosphatase (ALP) and osteocalcin (OCN) [79, 80, 83, 84, 85, 86]. These non‐coding RNAs regulate multiple pathways involved in osteoblast differentiation, ECM remodeling, and angiogenesis [83, 87, 88, 89]. For instance, lncRNAs such as MALAT1 and HOTAIR have been implicated in enhancing osteoblast differentiation by regulating transcription factors involved in the Wnt/β‐catenin and BMP‐Smad pathways [90, 91]. LncRNA H19 promotes osteogenesis by upregulating stromal cell‐derived factor‐1 (SDF‐1) via miR‐149, while an inhibition of miR‐182 leads to an increased bone mass by regulating osteoclastogenesis [92, 93]. Similarly, circRNAs such as circRNA‐23 525 act as competitive endogenous RNAs (ceRNAs), sponging miR‐30 to maintain the expression of osteogenic factors like ALP and OCN [94]. By leveraging EVs enriched with these regulatory molecules, researchers aim to refine therapeutic strategies that harness MSCs' regenerative potential while circumventing concerns related to immune rejection and uncontrolled differentiation. The application of EVs may offer a novel means in spinal fusion therapy. Nevertheless, several translational barriers remain. Scalable EV production with consistent quality and potency is technically challenging, and efficient delivery to the fusion site is not yet fully optimized. Moreover, most current evidence comes from animal studies, often not in aged models, and further validation in elderly populations is required.

### 3.4 Surgical and Biomechanical Optimization

Advancements in spinal instrumentation have significantly improved fusion rates by optimizing load distribution and minimizing perioperative complications.

Minimally invasive surgical (MIS) techniques for pedicle screw placement with additional lateral lumbar interbody fusion (LLIF) and MIS transforaminal lumbar interbody fusion (TLIF), offer advantages over traditional open approaches by reducing soft tissue trauma, blood loss, and recovery time [95, 96, 97]. Additionally, unilateral biportal endoscopy (UBE) has emerged as a novel technique in spinal surgery, providing a minimally invasive alternative for decompression and fusion procedures. UBE allows for improved visualization, reduced iatrogenic muscle injury, and precise neural decompression, enhancing surgical outcomes while preserving paraspinal muscle integrity [98, 99]. Studies have shown that UBE can lead to lower postoperative pain scores and faster functional recovery compared to conventional MIS approaches [100]. This is particularly beneficial for elderly patients with diminished physiological reserve. Furthermore, computational modeling and finite element analysis (FEA) are increasingly employed to design patient‐specific implants that balance mechanical rigidity with bone remodeling dynamics, addressing challenges such as osteoporosis‐related fragility [101]. In addition to these advancements, robotic‐assisted spine surgery and augmented reality (AR)‐guided navigation systems are being integrated into MIS techniques to enhance surgical precision and reduce intraoperative complications. Robotic‐assisted systems improve pedicle screw placement accuracy, reducing the risk of malposition‐related complications, while AR‐guided navigation enhances real‐time intraoperative visualization, aiding in complex spinal procedures [102, 103]. Another key development in this field is expandable interbody cages, which provide superior stability and facilitate bone ingrowth by maintaining optimal disc height and alignment [104, 105].

Together, these strategies above form the basis of current practice. In elderly patients, however, several unique considerations must be taken into account. Bone quality is frequently compromised by osteoporosis, reducing the reliability of autografts and impairing the integration of allografts or synthetic substitutes [106]. BMPs remain effective but carry increased risks of complications, necessitating careful dose adjustments [107]. Vascular impairment and cellular senescence limit the regenerative capacity of bone marrow–derived MSCs, while reduced MSC yield and function further diminish efficacy [108, 109]. Emerging cell‐free approaches, such as EVs, may partially circumvent these age‐related limitations, though clinical data remain sparse. Minimally invasive procedures are particularly advantageous in elderly patients by reducing blood loss and surgical trauma, while expandable cages offer improved stability in osteoporotic bone. A comparative overview of spinal fusion strategies is presented between elderly and non‐elderly patients in Table 1.

**TABLE 1:** Comparative overview of spinal fusion strategies in elderly vs. non‐elderly patients.

Category	Strategy	Effectiveness/outcomes (non‐elderly patients)	Considerations in elderly patients	References
Bone grafts/biomaterials	Autografts	Gold standard, high osteogenic potential, reliable fusion rates.	Limited by donor site morbidity, reduced graft quality in osteoporotic elderly	[26, 27, 28, 106]
	Allograft/Synthetic Substitutes (HA, β‐TCP, PEEK, DBM)	Provide osteoconductive scaffold, fusion rates are satisfactory.	Osteoporotic bone may impair integration	[26, 36, 37, 106]
Growth factors	BMP‐2/BMP‐7	Potent osteoinductive properties.	High complication risk (ectopic bone, swelling); dose adjustment needed in elderly	[26, 59, 107]
	VEGF/FGF‐2	Promote angiogenesis, enhance bone formation.	Elderly patients benefit due to impaired vascularization	[68, 69, 70, 109]
Cell therapies	MSCs	Differentiate into osteoblasts; enhance osteogenesis and immunomodulation.	Cell senescence and lower MSC yield reduce efficacy in elderly.	[71, 72, 73, 108]
	EVs	Deliver miRNAs, lncRNAs, and circRNAs to enhance osteogenesis and avoid immune rejection.	Potentially overcome age‐related limitations, but clinical data lacking.	[78, 79, 80, 81, 82]
Surgical techniques	MIS	Reduced tissue trauma and blood loss, faster recovery, lower infection risk.	Particularly beneficial for elderly with comorbidities	[95, 96, 97, 98, 99]
	Expandable cages	Maintain disc height and alignment, promote bone ingrowth.	Osteoporotic bone requires enhanced stability; expandable cages reduce subsidence.	[104, 105, 110]

Abbreviations: BMPs, bone morphogenetic proteins; circRNAs, circular RNAs; DBM, demineralized bone matrix; EVs, extracellular vesicles; FGF‐2, fibroblast growth factor‐2; HA, hydroxyapatite; lncRNAs, long non‐coding RNAs; miRNAs, microRNAs; MIS, minimally invasive surgery; MSC, mesenchymal stromal cell; PEEK, polyetheretherketone; VEGF, vascular endothelial growth factor; β‐TCP, β‐tricalcium phosphate.

## 4 Innovative Approaches for Elderly Patients

Elderly patients present unique challenges in spinal fusion due to age‐related declines in osteoblastogenesis, stem cell senescence, and comorbidities such as diabetes and osteoporosis. Novel therapeutic strategies targeting these pathophysiological mechanisms are essential to improve fusion outcomes in this population.

### 4.1 Targeting Cellular Senescence and Oxidative Stress

Aging is associated with a progressive accumulation of senescent osteoblasts and mesenchymal progenitors, resulting in impaired bone formation and delayed fracture healing [19]. Cellular senescence is driven by an upregulation of the p16INK4a and p21 pathways, leading to a senescence‐associated secretory phenotype (SASP) that disrupts the bone microenvironment by increasing pro‐inflammatory cytokines, such as IL‐6 and TNF‐α [111, 112, 113, 114, 115]. Senolytic agents, including dasatinib and quercetin, have emerged as promising interventions capable of selectively eliminating senescent cells, thereby restoring osteogenic differentiation potential and mitigating chronic inflammation at the fusion site [116, 117]. Preclinical studies also suggest that these therapeutic agents can enhance bone formation capacity by reducing the adverse osteogenic effects of biomaterial‐related infections [118].

Mitochondrial dysfunction is a hallmark of aging, contributing to increased oxidative stress and impaired ATP production, which in turn accelerates MSCs' senescence and compromises their osteogenic potential [119, 120]. Reactive oxygen species (ROS) accumulation is another manifestation of aging [120, 121]. Recent advances in mitochondria‐targeted therapeutics, such as Mitoquinone (MitoQ), elamipretide (SS‐31), have demonstrated potential in scavenging excessive ROS, restoring mitochondrial membrane potential, and enhancing MSCs' survival under hypoxic conditions [122, 123]. These compounds not only improve mitochondrial bioenergetics but also modulate key transcriptional regulators, which govern mitochondrial biogenesis and oxidative phosphorylation, ultimately facilitating bone formation. However, long‐term efficacy and clinical safety still need further study.

### 4.2 Epigenetic and Gene‐Editing Interventions

Epigenetic modifications, including DNA methylation, histone deacetylation, and chromatin remodeling, are increasingly recognized as pivotal factors contributing to the decline of osteogenic potential with age. DNA methylation dysfunction of osteogenic gene promoters, such as Runt‐related transcription factor 2 (RUNX2) and Osterix (OSX), coupled with histone deacetylation‐driven transcriptional repression, leads to decreased osteoblast differentiation and bone formation [124, 125, 126]. Pharmacological interventions such as histone deacetylase (HDAC) inhibitors (e.g., valproic acid, trichostatin A) and DNA demethylating agents (e.g., 5‐azacytidine, decitabine) have demonstrated potential in reversing these epigenetic silencing effects, thereby restoring osteogenic gene expression [127, 128]. These studies provide an option to facilitate bone formation in the elderly.

CRISPR‐Cas9‐based gene editing presents an innovative approach for precise modulation of pro‐osteogenic pathways critical for bone formation and fusion. Recent advancements have enabled targeted activation of the Wnt/β‐catenin and BMP‐Smad signaling cascades by selectively editing regulatory elements or removing inhibitory epigenetic modifications [129]. CRISPR‐mediated knockout of LRP5 has been shown to reduce Wnt signaling activity, thereby decreasing vertebral bone volume and bone mineralization [130]. Additionally, the deletion of Wnt signaling pathway inhibitors, Sclerostin (SOST) and Dickkopf‐1 (DKK1), has demonstrated improved bone mass and fusion efficiency [131, 132]. Through gene‐editing strategies combined with nanoparticle‐based delivery systems, it holds significant insight for elderly patients suffering from osteoporosis and spinal degeneration. Meanwhile, gene editing and epigenetic modifications pose significant challenges, including ethical concerns regarding human genome manipulation and the risk of unintended off‐target mutations that may lead to unpredictable biological consequences [133].

### 4.3 Advanced Biomaterial Design for Osteoporotic Bone

Osteoporosis‐driven bone fragility necessitates biomaterials with highly specialized mechanical and biological properties to ensure both structural integrity and enhanced bone formation. Bioactive glass–ceramic composites, particularly those doped with osteogenic ions, such as silicon, have been shown to exert dual functions: promoting osteoblast differentiation while simultaneously inhibiting infection, and even improving bone angiogenesis [134, 135, 136, 137]. Strontium‐substituted bioglasses modulate autophagy and the Akt/mTOR signaling pathway, enhancing osteoporotic bone formation [138]. A new bone cement type that combines magnesium/strontium co‐substituted nano‐hydroxyapatite (n‐HA) with calcium phosphate dibasic and chitosan/gelatin polymers is found to have osteoinductive potential, stimulating ECM mineralization and differentiation of MC3T3‐E1 cells [139]. Additionally, the advent of 4D‐printed shape‐memory polymer scaffolds has introduced a new dimension to biomaterial engineering, allowing scaffolds to dynamically adapt to the evolving bone remodeling process. These smart scaffolds respond to physiological stimuli such as temperature, pH shifts, or enzymatic activity, enabling controlled degradation and real‐time mechanical adaptation to the bone formation environment. Moreover, they serve as advanced drug delivery systems by incorporating osteogenic agents such as teriparatide or BMP‐2, ensuring sustained and localized release to enhance osteogenesis [140]. Such customized biomaterials exhibit significant potential for personalized spinal fusion applications, particularly in elderly patients with compromised bone metabolism. However, their clinical translation is challenged by intricate manufacturing processes and high associated costs.

### 4.4 Immunomodulatory Strategies

Chronic low‐grade inflammation (“inflammaging”) is a hallmark of aging and a significant contributor to impaired bone homeostasis, primarily through sustained activation of osteoclastogenesis and suppression of osteoblastic activity [141, 142]. This prolonged inflammatory state is driven by an imbalance in pro‐inflammatory and anti‐inflammatory cytokines, with elevated levels of TNF‐α, IL‐6, and IL‐1β exacerbating bone resorption [141, 143]. Consequently, strategies aimed at immunomodulation have gained increasing attention for enhancing spinal fusion outcomes. The localized delivery of anti‐inflammatory cytokines, such as IL‐4 and IL‐13, using advanced hydrogel‐based carriers, has demonstrated potential in shifting the macrophage polarization from a pro‐inflammatory M1 phenotype toward a regenerative M2 phenotype [144, 145, 146]. This transition not only mitigates inflammatory‐mediated bone loss but also fosters a favorable osteogenic microenvironment. It offers a promising strategy for sustained immunomodulation and creates a pro‐regenerative niche, ultimately improving the success rates of spinal fusion in aging populations (Table 2).

**TABLE 2:** Innovative approaches to enhance spinal fusion for elderly patients.

Category	Strategy	Mechanism of action	Advantages	Challenges	References
Targeting senescence	– Senolytic agents (dasatinib, quercetin). – Mitochondria‐targeted antioxidants (MitoQ, SS‐31).	– Eliminate senescent cells, reduce oxidative stress, restore mitochondrial function.	– Enhance osteogenic differentiation, reduce inflammation.	– Long‐term safety and efficacy data lacking.	[116, 117, 122, 123]
Epigenetic/gene editing	– HDAC inhibitors (valproic acid, trichostatin A). – CRISPR‐Cas9 for Wnt/β‐catenin, BMP‐Smad pathways.	– Reverse epigenetic silencing, activate osteogenic genes.	– Restore osteogenic potential, precise gene editing.	– Ethical concerns, off‐target mutations.	[127, 128, 130, 133]
Advanced biomaterials	– Bioactive glass–ceramic composites and new bone cement type. – Strontium‐substituted bioglasses. – 4D‐printed shape‐memory polymer scaffolds.	– Promote osteoblast differentiation. – Adapt to bone remodeling.	– Enhance bone density, mechanical strength. – Inhibit infection. – Controlled drug delivery.	– Complex manufacturing, high cost.	[134, 135, 136, 137, 138, 139, 140]
Immunomodulation	– Anti‐inflammatory cytokines (IL‐4, IL‐13).	– Shift macrophage polarization (M1 to M2), reduce inflammation.	– Create pro‐regenerative niche, enhance osteoblast differentiation.	– Systemic side effects are unknown.	[144, 145, 146]

Abbreviations: HDAC, histone deacetylase; IL, interleukin; MitoQ, mitoquinone; SS‐31, elamipretide.

## 5 Current Focus and Controversies

### 5.1 Current Focus

#### 5.1.1 Balancing Osteogenesis and Complication Reduction

The dual challenge of promoting bone formation and implant ingrowth while minimizing complications (heterotopic ossification, infections, and inflammation) remains a central focus. For elderly patients, this balance is further complicated by age‐related comorbidities and reduced healing capacity. Recent studies emphasized the importance of controlled BMP delivery to avoid supraphysiological doses, which are linked to adverse effects such as ectopic bone formation and soft tissue swelling [26].

#### 5.1.2 Optimizing BMP Use in Elderly Patients

BMP‐2 and BMP‐7 are widely used in bone repair. A randomized controlled trial showed that BMP‐2 significantly improved spinal fusion for patients in terms of mid‐term (36 months after surgery) treatment. Yet their long‐term efficacy and safety for elderly patients were unclear [147]. Controversially, some researchers advocate for lower BMP doses to mitigate complications [107, 148], though this risks suboptimal osteogenesis in elderly patients.

#### 5.1.3 Cell Therapy Standardization

MSCs‐based therapies hold potential for elderly patients due to their immunomodulatory and osteogenic properties. However, heterogeneity in MSC sources (e.g., bone marrow vs. adipose tissue), dosing, and delivery methods complicates clinical translation [149]. Recent efforts focus on standardizing protocols, such as the International Society for Cellular Therapy (ISCT) guidelines for MSC characterization [150]. Nevertheless, the high production and delivery costs of MSC‐based approaches raise legitimate concerns about cost‐effectiveness in the context of spinal fusion. Such standardization is not only a technical requirement but also directly relevant to clinical outcomes. By ensuring that transplanted MSCs consistently meet defined criteria for viability, differentiation capacity, and immunomodulatory activity, the probability of successful graft integration and bone fusion increases [151, 152]. Uniform expansion and delivery methods help to optimize cell survival at the fusion site, thereby reducing variability in fusion rates between studies. Moreover, standardized dosing regimens and delivery carriers minimize risks of inflammatory reactions or ectopic tissue formation, lowering complication rates [153].

#### 5.1.4 Autograft Alternatives and Synthetic Biomaterials

Autografts are widely regarded as the gold standard in bone formation and tissue engineering due to their superior biocompatibility, osteogenic potential, and integration capabilities. However, the associated donor site morbidity, including postoperative pain, infection risks, prolonged recovery time and limited availability of harvestable tissue, has promoted the development of alternative solutions. In response, synthetic biomaterials have gained significant attention as promising substitutes and are being extensively researched to address these challenges [41, 55, 137] (Table 3).

**TABLE 3:** Current focus in enhancing spinal fusion for elderly patients.

Focus area	Key challenges	Recent advances	References
Balancing osteogenesis and complications	High BMP doses cause heterotopic ossification and inflammation.	Controlled BMP delivery via hydrogels and nanoparticles reduces systemic toxicity.	[26, 46, 47]
Optimizing BMP use	BMP‐2 efficacy vs. safety in elderly patients.	Lower BMP doses reduce complications but may compromise fusion.	[147]
Cell therapy	Heterogeneity in MSC sources and delivery methods.	Standardization of MSC protocols (e.g., ISCT guidelines) improves clinical outcomes.	[149, 150]
Autograft alternatives and synthetic biomaterials	Donor site morbidity, especially osteoporosis, limits autograft uses in elderly patients.	Synthetic biomaterials promote osteoconduction and angiogenesis, as promising substitutes.	[41, 55, 137]

Abbreviations: BMP, bone morphogenetic protein; ISCT, International Society for Cellular Therapy.

### 5.2 Key Controversies

#### 5.2.1 Autograft: Is the Gold Standard Obsolete?

Autografts are favored for their osteogenic potential, but elderly patients often lack sufficient viable bone. In a multicenter randomized trial of posterolateral lumbar fusion, patients received AttraX Putty on one side of the fusion trajectory and autograft on the contralateral side, serving as their own controls; after 1‐year follow‐up, fusion rates were comparable between autografts and biphasic calcium‐phosphate substitute (52% vs. 55%, *p* = 0.617), thereby reigniting debate over the necessity of autografts [154]. Critics argue that biomaterial success depends heavily on adjuvant therapies (e.g., BMPs), which may offset cost savings [155].

#### 5.2.2 Anti‐Osteoporosis Drugs: Benefit or Risk?

Bisphosphonates improve bone density but may suppress bone remodeling, potentially delaying fusion [156]. A study found that teriparatide significantly increased fusion rates at long‐term postoperative periods compared to bisphosphonates (OR 2.05, 95% CI 1.17–3.59, *p* = 0.01). However, long‐term teriparatide use is limited by cost and hypercalcemia risks [157].

#### 5.2.3 Ethics of Emerging Technologies

Gene‐editing technologies, such as CRISPR, and senolytic therapies have demonstrated considerable therapeutic potential in preclinical studies. However, their application raises ethical and safety concerns [158]. CRISPR‐mediated modifications of MSCs could theoretically enhance spinal fusion outcomes but carry the inherent risk of off‐target mutations, which may lead to unintended genetic alterations [159]. Likewise, while senolytic agents have shown promise in accelerating bone formation, their long‐term safety and efficacy in aging populations remain insufficiently characterized, necessitating further investigation [117, 118] (Table 4).

**TABLE 4:** Key controversies in spinal fusion for elderly patients.

Controversy	Arguments for	Arguments against	References
Autograft vs. allograft	Autografts are the gold standard for osteogenesis.	Biphasic calcium‐phosphate shows comparable fusion rates without donor morbidity.	[154, 155]
Anti‐osteoporosis drugs	Bisphosphonate and teriparatide enhance fusion rates in osteoporotic patients.	Bisphosphonate suppress bone remodeling in spinal fusion and long‐term teriparatide use is costly and may cause hypercalcemia.	[156, 157]
Emerging technologies	Gene‐editing (e.g., CRISPR) and senolytics show preclinical promise.	Ethical concerns and lack of long‐term safety data in elderly populations.	[117, 118, 158, 159]

Abbreviation: MSC, mesenchymal stromal cell.

## 6 Future Perspectives

As the field of spinal fusion continues to evolve, future advancements will rely on a multidisciplinary approach, integrating expertise from orthopedics, bioengineering, and regenerative medicine. Collaborative efforts among these disciplines will facilitate the development of more effective, minimally invasive, and patient‐specific treatment strategies. By leveraging cutting‐edge technologies, researchers and clinicians can address current limitations and optimize patient outcomes.

One of the most promising directions is the application of precision medicine, which tailors treatment strategies based on an individual's genetic, molecular, and physiological characteristics [160, 161]. Advances in genomics and proteomics allow for the identification of patient‐specific risk factors, enabling more accurate prognostic assessments and the customization of therapeutic interventions. This is particularly relevant for elderly patients, who often present with complex comorbidities and varying bone quality, necessitating highly individualized treatment plans.

The translation of novel technologies into clinical practice will further enhance spinal fusion outcomes. Artificial intelligence (AI) has the potential to revolutionize surgical planning by predicting postoperative outcomes and assisting in decision‐making. Moreover, AI‐driven image analysis can improve preoperative assessments, leading to more precise implant placement and reducing the risk of complications [162]. In elderly patients, AI may be particularly valuable for integrating complex variables such as osteoporosis severity, comorbidities, and polypharmacy into personalized risk models [163]. By stratifying patients according to biological age and surgical tolerance, AI‐based tools could help optimize the choice of fusion techniques, implants, and perioperative care. In this way, AI not only facilitates technical precision but also addresses the unique challenges of elderly low back pain, supporting individualized treatment pathways and improving both safety and functional recovery.

Another promising avenue is nanotechnology, which holds significant potential in drug delivery systems and bone formation [164]. The development of bioactive nanoparticles for targeted drug release and nanostructured scaffolds for bone formation could greatly enhance fusion efficacy and patient recovery [165].

To ensure the clinical applicability of these advancements, large‐scale clinical trials are essential. Rigorous, well‐designed studies focusing on elderly populations will provide critical insights into the safety, efficacy, and long‐term benefits of novel treatments.

**FIGURE 2:**
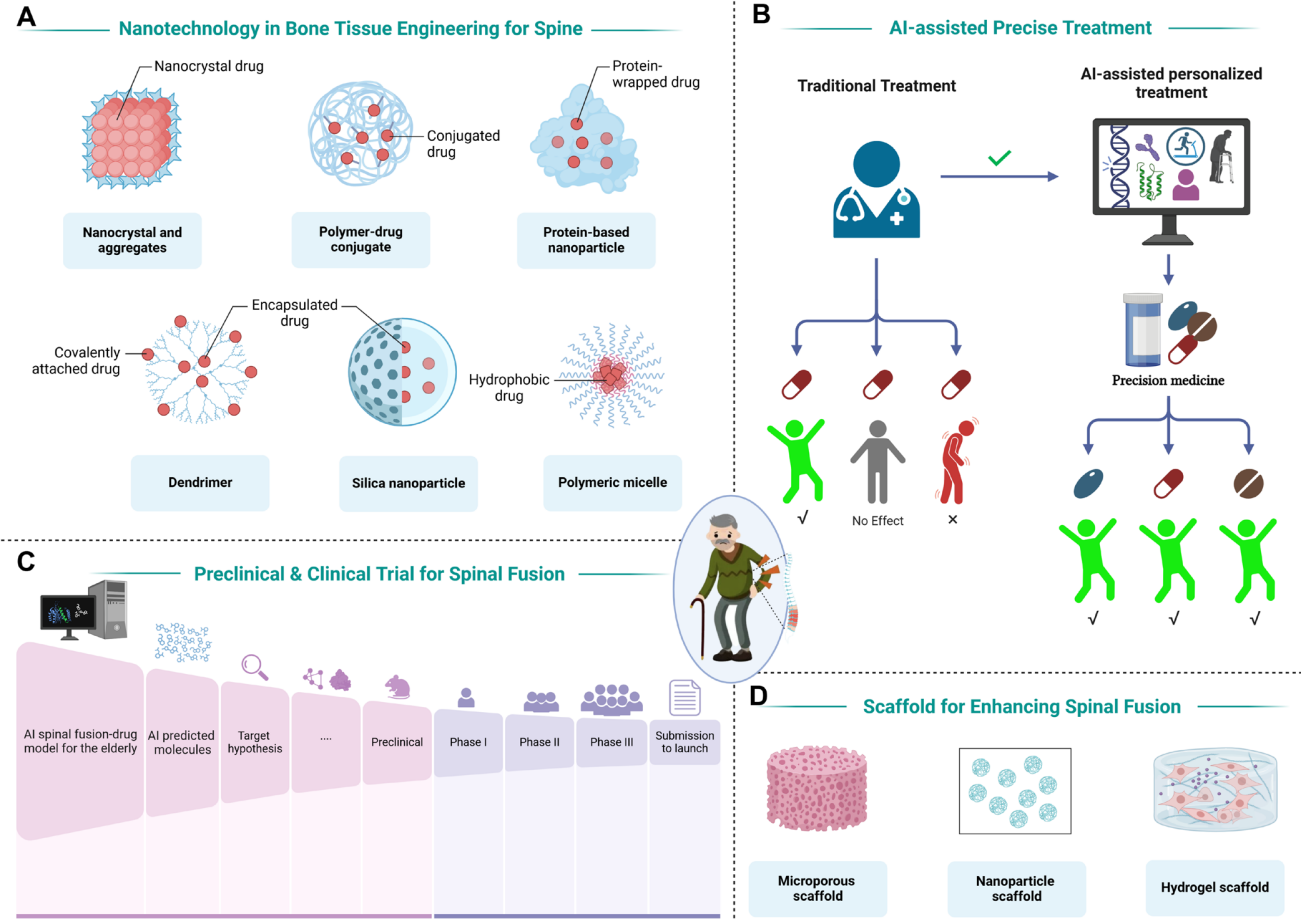
Future perspectives in spinal fusion: Integration of advanced technologies and multidisciplinary approaches. (A) Nanotechnology in bone formation for the spine. (B) AI‐assisted precise treatment. (C) Preclinical and clinical trials for spinal fusion. (D) Scaffold for enhancing spinal fusion.

Moving forward, the integration of emerging technologies with evidence‐based clinical approaches will be key to optimizing spinal fusion outcomes. Continued interdisciplinary collaboration, precision medicine initiatives, and robust clinical validation will pave the way for safer, more effective, and patient‐centric treatment (Figure 2).

## 7 Conclusion

In conclusion, although spinal fusion continues to represent an important surgical option for low back pain, its application in elderly patients remains particularly challenging due to compromised bone quality, reduced regenerative capacity, and the burden of comorbidities. Advances in biomaterials, growth factor delivery systems, and cell‐based therapies have significantly improved fusion outcomes by enhancing osteogenesis and modulating the inflammatory microenvironment. Additionally, innovations in minimally invasive surgical techniques, computational modeling, and gene‐editing approaches offer personalized treatment strategies, particularly for elderly patients with osteoporosis and impaired bone formation. Despite these advancements, challenges such as optimizing biomaterial integration, mitigating inflammatory responses, and ensuring long‐term stability remain. Future research should focus on multi‐modal therapeutic strategies that integrate precision medicine, AI, nanotechnology, regenerative biology and advanced biomaterial engineering to achieve enhanced spinal fusion outcomes while minimizing complications.

## Author Contributions

S.C. and B.G. conceived and designed the work; S.C. performed the original writing; S.C., B.G. and Z.L. contributed to the writing and editing; S.F.B., S.H. and C.E.A. provided problems encountered in clinical treatment; S.C. and B.G. decided the final version. S.C. created the graphic arts and tables. All authors have read and agreed to the published version of the manuscript.

## Consent

The authors have nothing to report.

## Conflicts of Interest

B.G. is a member of the editorial board of JOR Spine and all other authors declare no conflicts of interest.

## Data Availability

All the data used in this research are available on request from the first and corresponding author.
